# 1673. Time to positivity of blood cultures in a pediatric hospital: more tools to reconsider empirical treatment in suspected infections.

**DOI:** 10.1093/ofid/ofad500.1506

**Published:** 2023-11-27

**Authors:** Joam C Andrade-Fernandez, Ivan Felipe Gutierrez-Tobar, Juan Pablo Londono-Ruiz

**Affiliations:** STAPHYLORED COLOMBIA, bogota, Distrito Capital de Bogota, Colombia; Clínica Infantil Santa Maria del Lago y Clinica Infantil Colsubsidio, Bogota, Distrito Capital de Bogota, Colombia; Clinica Infantil Colsubsidio, Staphylored Colombia, Bogota, Distrito Capital de Bogota, Colombia

## Abstract

**Background:**

Blood cultures are essential for diagnosing bacterial infections. Positivity time (TP) measures the time from incubation to bacterial growth detection. Understanding TP for different pathogens and age groups can guide empirical treatment duration. Our aim is to describe microbiological characteristics of blood culture isolates by TP, causative germ, and patient age.

**Methods:**

This retrospective and observational study analyzed all blood culture isolates obtained from a pediatric reference center in Colombia between 2015 and 2021, using the automated BD Phoenix™ detection system. Data was extracted from WHONET and analyzed for average positivity time, patient age, and microorganism type.

**Results:**

A total of 1258 positive bottles were analyzed: 32.9% [414] were Gram-negative, 65.5% [824] were Gram-positive, and 1.6% [19] were yeast. The overall median positivity time was 14.5 [10.9-20.6] hours, for Gram-positive organisms, the median growth time was 17.2 [13.2-21.6] hours, and for Gram-negative organisms it was 10.8 [8.9-14.1] hours [p=< 0.0001]. 65.7% of Gram-negative organisms were identified within the first 12 hours, compared to 17.7% of Gram-positive organisms. (Figure 1). The pathogen with the fastest median growth time was S. agalactiae with 8.3 [6.7-10.3] hours [n=11], followed by K. pneumoniae with 9.5 [8.3-10.9] hours and S. pyogenes with 9.9 [7.7-12.1] hours [n=9]. Among clinically significant bacterial microorganisms with slower median positivity times were Haemophilus influenzae with 22.7 [14.8-23.9] hours [n=9], followed by coagulase-negative Staphylococcus species with a median of 19.6 [17.1-23.2] hours, then non-fermenting Gram-negative bacteria with a median of 16.4 [13.1-23.2] hours, and Salmonella spp with 16.3 [13.7-28.3] hours [n=22].

Distribution of positivity according to time intervals (Gram positive vs. Gram negative)

**Figure:**
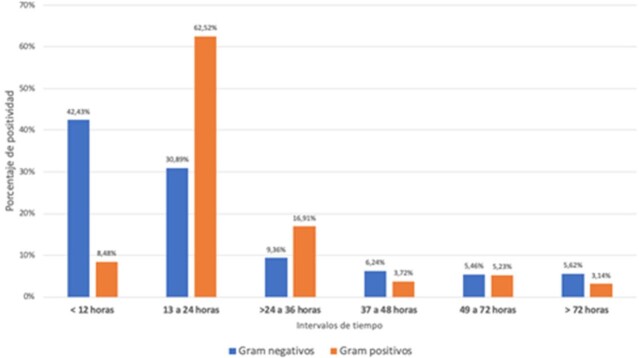

Proportion of microorganisms isolated according to age group

**Figure:**
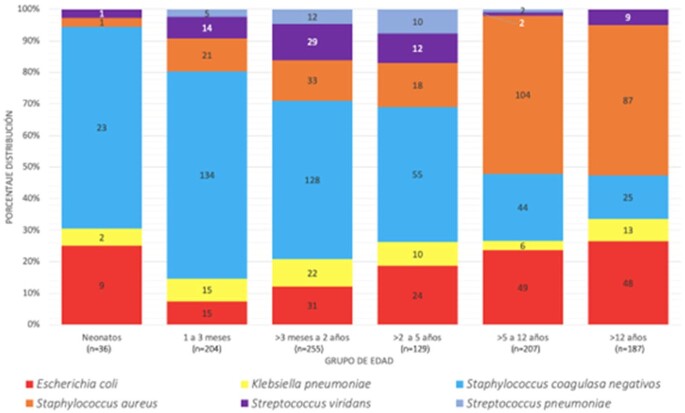

Positivity time in hours according to different groups of organisms

**Figure:**
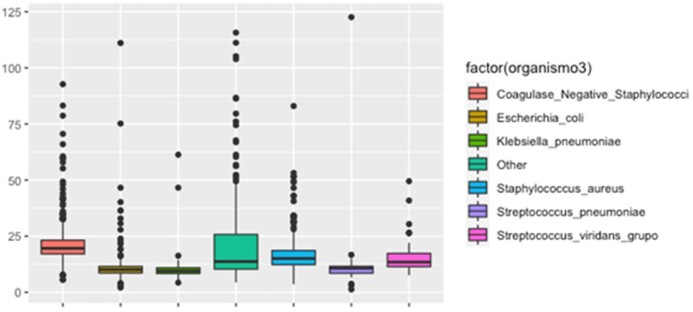

**Conclusion:**

Based on the study findings, it can be concluded that in pediatric patients with suspected bloodstream infection, empiric antimicrobial regimens can be shortened to 24-36 hours. The results of the study support this conclusion by demonstrating that the time to blood culture positivity was typically less than 24 hours. However, it would be beneficial to include additional data on the time of positivity associated with severity and clinical outcomes in pediatric patients to further support this conclusion.

**Disclosures:**

**All Authors**: No reported disclosures

